# Dynamic Population of Gut Microbiota as an Indicator of Inflammatory Bowel Disease 

**DOI:** 10.52547/ibj.3772

**Published:** 2022-11-20

**Authors:** Afsaneh Salimi, Amin Sepehr, Hossein Ajdarkosh, Shadi Aghamohammad, Maliheh Talebi, Mahdi Rohani, Mohammad Reza Pourshafie

**Affiliations:** 1 Department of Microbiology, Pasteur Institute of Iran, Tehran, Iran;; 2 Gastrointestinal and Liver Disease Research Center, Firoozgar Hospital, Tehran University of Medical Sciences, Tehran, Iran;; 3 Department of Microbiology, School of Medicine, Iran University of Medical Sciences, Tehran, Iran

**Keywords:** Inflammatory bowel disease, Microbiota, Real-time polymerase chain reaction

## Abstract

**Background:**

Inflammatory bowel disease is a chronic inflammatory disease of the gastrointestinal tract. The gut microbiota is an important factor in the pathogenesis of IBD. Due to a link between the gut microbiota and IBD, studying microbiota changes using an accurate, sensitive and rapid method for detection of the disease seems necessary. This study aimed to compare the composition of gut microbiota in three groups of people, including IBD patients, CIBD, and healthy groups.

**Methods:**

For this study, 45 stool samples (15 from each group) were collected. Using real-time PCR, the abundance of 11 bacterial 16S rRNA gene sequences was examined.

**Results:**

In the IBD group, the number of three bacterial phyla, including Firmicutes, Actinobacteria, and Bacteroidetes, decreased (*p* < 0.01, *p* < 0.01, and *p* < 0.001, respectively), while the population of γ-Proteobacteria increased significantly (*p* < 0.0001). In the CIBD group, the number of Actinobacteria enhanced (*p* < 0.01), but that of Bacteroidetes and Firmicutes decreased (*p* < 0.01, and *p* < 0.05, respectively).

**Conclusion:**

Findings of this study indicate that decrease in Firmicutes and increase in γ-Proteobacteria could be used as an indicator of IBD instead of employing invasive and costly detection methods such as colonoscopy and other tests.

## INTRODUCTION

Inflammatory bowel disease is a chronic inflammatory disease of the gastrointestinal tract, that results from impaired interaction between the gut immune system and the gut microbiota^[^^1^^,^^2^^]^. CD and UC are two main types of IBD that affect the large and small intestines and have the unique microbiota composition pattern^[^^1^^]^. In CD, inflammation occurs in any part of the gastrointestinal tract, whereas in UC, inflammation happens in the mucosal layer of the colon. The clinical features of IBD vary depending on the site and severity of inflammation, as well as the status of the disease in different individuals^[^^2^^]^.

While the exact cause of IBD remains unknown, several factors, including host’s genetics, immune system function, environmental factors, and gut microbiota have been shown to play important roles in development of the disease. In addition, multiple epidemiological factors, such as birth status (cesarean section), breastfeeding, smoking, health conditions, infections, antibiotics, diet, and sleep patterns are thought to cause IBD and also microbial changes in the body, indicating the important role of the gut microbiota in the pathogenesis of IBD^[^^3^^-^^6^^]^.

The intestinal microbiota is a large collection of microbes in the body, accounting for 10^12^ cells/g of the luminal contents of the intestine. In the gastrointestinal tract of healthy people, there are generally five bacterial phyla, including two main phyla (Firmicutes and Bacteroidetes) along with three other phyla (γ-Proteobacteria, Actinobacteria, and Verrucomicrobia)^[^^7^^]^. The gut microbiota has many significant functions in the host by inducing symbiosis. These functions include metabolism and synthesis of nutrients, especially vitamins K and B, the tropism of mucous membranes, metabolism of drugs and toxins, and protection against pathogens. In fact, the gut microbiota forms a complex and extremely important structure that acts as a link between the host and the environment. This intestinal barrier regulates the interaction between bacteria and host cells and modulates the uptake of nutrients^[^^8^^]^.

In IBD patients, the composition and function of the gut microbiota change^[^^9^^,^^10^^]^. The microbial taxonomic profile may differ from patient to patient, making it difficult to determine whether specific microbial species or strains are involved in disease development and progression^[^^11^^]^. The abundance of γ-Proteobacteria, such as *Enterobacteriaceae*, is increasing in IBD patients, whereas the proportion of Firmicutes such as *Lactobacillus* and *Faecalibacterium*, which have a protective effect on the gastrointestinal tract, is decreasing^[^^12^^-^^14^^]^. Evidence has shown that diversity of the gut microbiota, as well as the known beneficial bacteria such as *Faecalibacterium prausnitzii*, *Roseburia intestinalis*, and other butyrate producers decrease in IBD patients^[^^15^^]^.

Detection of IBD is challenging in terms of cost and side effects of methods. As mentioned earlier, changes in the gut microbiota could serve as an indicator of the progression of IBD. On the other hand, real-time PCR is an inexpensive and non-invasive method, compared to previously used approaches such as colonoscopy, for detecting IBD. Given these reasons, in this study, real-time PCR was used for 11 bacterial groups to identify alterations in the intestinal microbiota of IBD and CIBD patients compared to the healthy ones.

## MATERIALS AND METHODS

**Sample collection**

In this study, 45 stool samples were collected from three groups, including IBD patients, CIBD, and healthy individuals with a mean age of 33.5 years, ranging from 18 to 42 years. The first group (n = 15) consisted of patients with IBD who had been diagnosed with either UC (n = 8) or CD (n = 7). A specialized gastroenterologist diagnosed the cases based on clinical symptoms, as well as accepted radiological and paraclinical findings. The patients of this group suffered from painful complications such as diarrhea, bloody stools, abdominal pain, heartburn, and weakness. The second group (n = 15) were CIBD patients who were in remission, and whose disease was controlled by medication, i.e. mesalazine for all patients and thiopurine for some cases, depending on the severity of the disease. The last group (control group) included healthy individuals (n = 15), with inclusion criteria including normal body mass index, no history of gastrointestinal disease, no use of antibiotics in four weeks before sampling, no special diet, and no pregnancy. 

**DNA extraction**

DNA was extracted from the stool samples using the FavorPrep Stool DNA Isolation Mini Kit (Taiwan) in accordance with the manufacturer's instructions. The stool samples (200 mg) were mixed with 20 μl of proteinase K and 300 μl of lysis buffer in a bead tube, and the steps were continued according to the protocol of the extraction kit. The concentration and purity of DNA were measured using a NanoDrop 1000 UV-Vis spectrophotometer (BioTek, USA) and stored at -20 ºC until real-time PCR analysis.

**Amplification efficiency of real-time PCR**

Real-time PCR amplification efficiency was determined using a standard curve. At first, the extracted DNA of 10 stool samples was mixed in a microtube. Then serial dilutions (10^-1^-10^-5^) of this DNA were prepared and used to generate the standard curve for determination of slope, y-intercept, and correlation coefficient values.

**Determining the gut microbiota composition**

Investigation of the bacterial 16S rRNA genes in the stool sample was carried out based on the primers listed in Table 1 by using an ABI Step One Plus detection system (Applied Biosystems, USA). The real-time PCR amplifications were performed in 20 μl of reaction volume using 12 µl of 2× SYBR Green Master Mix (Amplicon, Denmark), 0.5 μmol/l of each primer, 3 µl of template DNA, and nuclease-free water to a final volume of 20 μl. Amplification included one cycle at 95 ºC for 15 minutes for initial denaturation, followed by 40 cycles of denaturation at 95 ºC for 15 seconds and primer annealing at 60 ºC for 60 seconds. Data were analyzed using RQ = 2^-ΔΔCt^ equation, in which the readings were normalized with all bacterial genes^[^^16^^]^. In this method, the Ct values of the target bacterium were normalized with all bacterial genes, and their comparison was evaluated using the comparative fold change. 

**Table 1:** Sequences of oligonucleotide primers used in this study

**Target bacterial**	**Sequence (5'–3')**	**Amplicon** **size (bp)**	**References**
All bacteria	F: TCCTACGGGAGGCAGCAGT	466	
	R: GGACTACCAGGGTATCTATCCTGTT		
Actinobacteria	F: TACGGCCGCAAGGCTA	300	
	R: TCATCCCCACCTTCCTCCG		
γ-Proteobacteria	F: TCGTCAGCTCGTGTAGTGA	154	
	R: CGTAAGGGCCATGATG		
Firmicutes	F: TGAAACTAAAAGGAATTGACG	155	
	R: ACCATGCACCACCTGTC		
Bacteroidetes	F: CRAACAGGATTAGATACCCT	204	30
	R: GGTAAGGTTCCTCGCGTAT		
*Lactobacillus*	F: TGGATGCCTTGGCACTAGGA	92	
	R: AAATCTCCGGATCAAAGCTTACTTAT		
*Bifidobacterium*	F: GGGTGGTAATGCCGGATG	278	
	R: TAAGCCATGGACTTTCACACC		
*Enterobacteriaceae*	F: CATTGACGTTACCCGCAGAAGAAGC	195	
	R: CTCTACGAGACTCAAGCTTGC		
*Enterococcus* *faecalis*	F: AACCTACCCATCAGAGGG	360	
	R: GACGTTCAGTTACTAACG		
*Clostridium * *clostridioforme*	F: AATCTTGATTGACTGAGTGGCGGAC	148	
	R: CCATCTCACACTACCGGAGTTTTTC		
*Roseburia*	F: TACTGCATTGGAAACTGTCG	230	
	R: CGGCACCGAAGAGCAAT		
*Faecalibacterium prausnitzii*	F: AGAGTTTGATCATGGCTCAG	191	
	R: GGTTACCTTGTTACGACTT		

**Statistical analysis**

All data were expressed as mean ± standard deviation. Differences in gut microbiome composition between the groups were determined using a one-way analysis of variance (ANOVA). All comparisons were analyzed by the 2^-ΔΔCt^ method. GraphPad Prism 8.0.2 was used for statistical analysis of the data. Differences were considered statistically significant at a value of *p* < 0.05. 

## RESULTS

**Comparison of gut microbiota composition in the study groups**

**Fig. 1:**
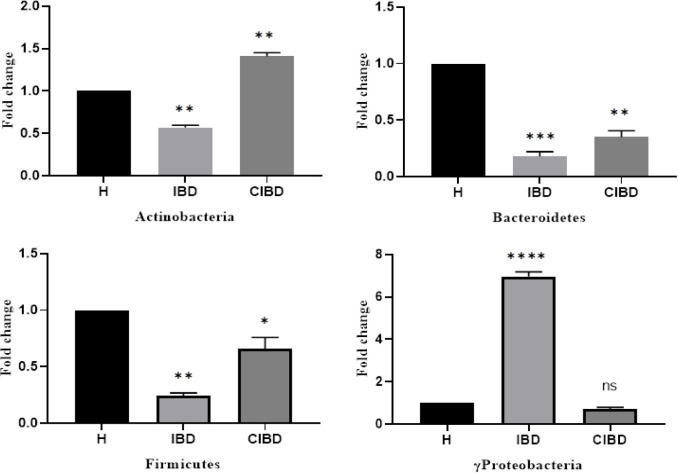
Differences in the abundance of bacterial phyla in IBD patients and CIBD compared to the control group (^*^p < 0.05, ^**^p < 0.01, ^***^p < 0.001, and ^****^p < 0.0001). H, healthy; ns, not significant

**Fig. 2:**
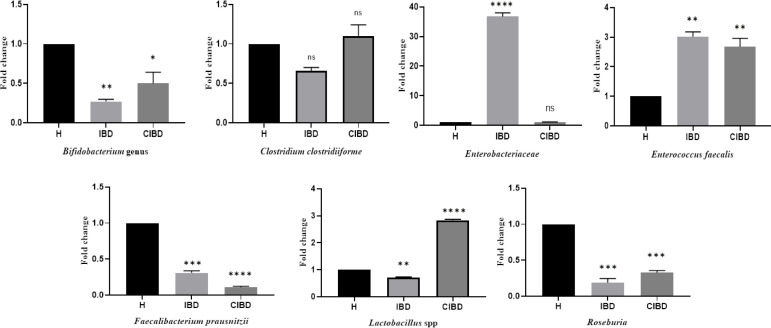
Differences in bacterial abundance in IBD patients and CIBD compared to control group. Statistical analysis was performed using one-way ANOVA test (^*^p < 0.05, ^**^p < 0.01, ^***^p < 0.001 and ^****^p < 0.0001). ns, not significant; H, healthy

Results of real-time PCR showed that the bacterial composition of the intestine was different in all groups of individuals. Using the relative method, changes in the intestinal microbiota of the three groups were observed. The number and diversity of the intestinal microbiota significantly reduced in IBD patients compared to the healthy group. The number and bacterial variations were also lower in the CIBD compared to the healthy group. The abundance of the three bacterial phyla, i.e. Firmicutes, Actinobacteria, and Bacteroidetes, decreased in the IBD group compared to the healthy group, while that of γ-Proteobacteria increased (Fig. 1). In contrast, in the CIBD group, the abundance of Actinobacteria increased, and the composition of Bacteroidetes and Firmicutes decreased. In both the IBD and CIBD groups, population of *Clostridium clostridioforme* did not significantly changed compared to the healthy group (Fig. 2).

**Comparison of gut microbiota composition of CD and UC patient subgroups**

**Fig. 3:**
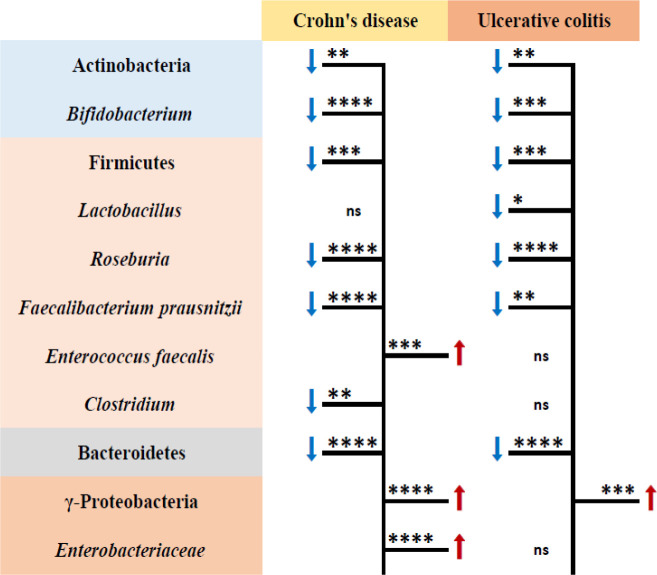
Gut microbiota associated with CD and UC compared to the control. Statistical analysis was performed with  one-way ANOVA test (^*^p < 0.05, ^**^p < 0.01, ^***^p < 0.001, and ****p < 0.0001). Increase **⬆** and decrease **⬇** in abundance of bacterial phyla; ns: not significant

According to the results of real-time PCR, the total amount of bacteria in the CD group was lower than that of the UC group. Comparison of CD and UC groups with the healthy group showed different results in some bacterial groups, including *Lactobacillus*, *Enterococcus faecalis*, *Clostridium*, and *Enterobacteriaceae* (Fig. 3). Comparing the CD-cured and UC-cured groups with the healthy group indicated an increase in the abundance of Actinobacteria (*p* < 0.0001), γ-Proteobacteria (*p* < 0.0001), and Bacteroidetes (*p* = 0.02) in the UC-cured group. Also population of Firmicutes increased in the CD-cured group compared to the healthy group (*p* = 0.03).

## DISCUSSION

Alteration in the composition of the gut microbiota may influence the pathogenesis and etiology of IBD^[^^17^^]^. In this study, the real-time PCR technique and the relative method were used to compare the composition of the gut microbiota in IBD and CIBD groups with healthy group.

The results of this study showed that the composition of the gut microbiota in IBD patients was significantly different from the healthy group and CIBD. The findings of gene amplification of all bacteria also demonstrated that the total amount of bacteria in the gut of IBD patients reduced compared to the healthy group. In IBD patients, the three major bacterial phyla of the gut; Bacteroidetes, Firmicutes, and Actinobacteria, reduced. There are conflicting reports on changes in the Bacteroidetes population in the IBD group. A study in the Netherlands reported an increase in the Bacteroidetes group^[^^18^^]^, whereas a decrease was observed in the Bacteroidetes population of IBD, in a study conducted in India^[^^19^^]^. This discrepancy may be due to differences in environmental and population conditions. Our results of the Bacteroidetes population in both CD and UC groups displayed that decrease in the Bacteroidetes was more evident in CD group than in the UC group. However, in the CIBD group, the population of Bacteroidetes was significantly (*p* < 0.01) lower than in the healthy group, suggesting that it takes longer time for the gut microbiota, including Bacteroidetes, to return to the normal population. Firmicutes phyla examined in this study to compare patients with healthy individuals include *Lactobacillus*, *Faecalibacterium*, *Roseburia*, *Clostridium*, and *Enterococcus*. In our study, all of these bacterial groups were examined in the stool samples of the three study groups. The results showed that the population of *Lactobacillus* decreased in the two groups CD and UC compared to the healthy group. It has also been reported that *lactobacilli*, as one of the most important probiotics, have ability not only to inhibit pro-inflammatory cytokines but also to balance the gut microbiota^[^^20^^]^. Our study disclosed that the *lactobacilli* load was very low in the UC group, which in turn could set the stage for inflammation. On the other hand, in the treated group, especially in the cured UC, the amount of *lactobacillus* was higher compared to the healthy group, suggesting that a change in medication and diet could increase the *lactobacillus* population again. The abundance of *Faecalibacterium* and *Roseburia* in the patients with IBD, as well as in the treated patients, was also significantly lower compared to the healthy group. *F. prausnitzii* and its metabolites may have a significant effect on the prevention of IBD by increasing bacterial diversity. *F. prausnitzii* could be influenced by increasing short-chain fatty acid-producing bacteria, decreasing level of TNF-α, and also the population of γ-Proteobacteria^[^^21^^]^. In one study, it was found that the reduction of *F. prausnitzii* in the CD subgroup could be the result of oxidative stress caused by intestinal inflammation and the reduction of antioxidant biosynthetic pathways^[^^22^^]^. *Roseburia* also plays an important role in inhibiting inflammation by enhancing the differentiation of Treg cells and anti-inflammatory cytokines such as TSLP, IL10, and TGF-β^[^^23^^]^. Reduction of these beneficial bacteria in the gut of patients with IBD led to inflammation in the gastrointestinal tract, which pathobiont bacteria can exploit and proliferate.

In the current study, with the reduction of Firmicutes and Bacteroidetes in the IBD group, the abundance of γ-Proteobacteria, one of the most important classes of Proteobacteria, enhanced. The increase in γ-Proteobacteria in the CD group was greater than that of the UC group, and a significant difference was found between the two subgroups. A survey has shown that the population of Proteobacteria in IBD has been proposed as a potential diagnostic marker for dysbiosis of the intestinal microbiota^[^^22^^]^. The abundance of *Enterobacteriaceae* in the IBD group was significantly different from the CIBD and healthy groups and was significantly higher in the CD group than in the UC group. This bacterial group may increase intestinal permeability and inflammation by stimulating the secretion of cytokines IL-8, TNF-α, IL -1β, and the destruction of mucosal junctions^[^^24^^]^. The findings of a study similar to our results showed an increase in *Enterobacteriaceae* population alone in the CD group, whereas no change was seen in the UC group^[^^18^^]^.

In general, microbial changes occur more frequently in patients with CD than in patients with UC. The results of this study are in agreement with previous studies that identified intestinal inflammation as one of the main factors responsible for the differences in the microbiome in CD and UC patients^[^^2^^,^^25^^]^. On the other hand, the γ-Proteobacteria and *Enterobacteriaceae* in the treated group were not significantly different from those in the healthy ones. Altogether, each of the subgroups CD and UC has its own microbial population in both the IBD patient group and CIBD group. Thus, the cured CD and UC subgroups were similar only in terms of the composition of *F. prausnitzii*.

The present study showed that important groups of bacteria, which play an important role in causing or inhibiting inflammation, belong two phyla Firmicutes and Proteobacteria; hence, the changes in population of these two phyla can be used as diagnostic markers. In addition, real-time PCR can be used as an accurate, sensitive, and non-invasive molecular method compared to the conventional IBD diagnostic methods to identify this disease.

## DECLARATIONS

### Acknowledgements

Authors appreciate the co-operation of all personnel in the Bacteriology Laboratory at the Pasteur Institute of Iran, Tehran.

### Ethical statement

The study protocol was approved by the Ethics Committee of the Pasteur Institute of Iran, Tehran (ethical code: IR.PII.REC.1398.060). Signed informed consent was obtained from all participants.

### Data availability

The datasets generated during and/or analyzed during the current study are available from the corresponding author on reasonable request.

### Authors’ Contributions

AS: designed the research, carried out sampling, lab work, statistical analysis, and data collection, and wrote the final manuscript; AS: contributed to lab works and data analysis; HA: developed the original idea, provided materials, and supervised the work; SA: contributed to lab works and data analysis; MT: developed the original idea, provided materials, and supervised the work; MR: developed the original idea, provided materials, and supervised the work; MRP: developed the original idea, provided materials, and supervised the work. 

### Conflict of interest

None declared.

### Funding/support

This work was supported by the Pasteur Institute of Iran (grant number BP-9532). 
